# Acute balenine supplementation in humans as a natural carnosinase-resistant alternative to carnosine

**DOI:** 10.1038/s41598-023-33300-1

**Published:** 2023-04-20

**Authors:** Sarah de Jager, An Vermeulen, Siegrid De Baere, Thibaux Van der Stede, Eline Lievens, Siska Croubels, Ralf Jäger, Martin Purpura, Jan G. Bourgois, Wim Derave

**Affiliations:** 1 Department of Movement and Sports Sciences, Ghent University, Watersportlaan 2, 9000 Ghent, Belgium; 2 Department of Bioanalysis, Ghent University, Ottergemsesteenweg 460, 9000 Ghent, Belgium; 3 Department of Pathobiology, Pharmacology and Zoological Medicine, Ghent University, Salisburylaan 133, 9820 Merelbeke, Belgium; 4 Department of Nutrition, Exercise and Sports, Copenhagen University, Nørre Allé 51, 2200 Copenhagen, Denmark; 5 Increnovo LLC, 730 E. Carlisle Avenue, Whitefish Bay, WI 53217 USA

**Keywords:** Nutrition, Metabolism

## Abstract

Balenine possesses some of carnosine’s and anserine’s functions, yet it appears more resistant to the hydrolysing CN1 enzyme. The aim of this study was to elucidate the stability of balenine in the systemic circulation and its bioavailability in humans following acute supplementation. Two experiments were conducted in which (in vitro) carnosine, anserine and balenine were added to plasma to compare degradation profiles and (in vivo) three increasing doses (1–4–10 mg/kg) of balenine were acutely administered to 6 human volunteers. Half-life of balenine (34.9 ± 14.6 min) was respectively 29.1 and 16.3 times longer than that of carnosine (1.20 ± 0.36 min, *p* = 0.0044) and anserine (2.14 ± 0.58 min, *p* = 0.0044). In vivo, 10 mg/kg of balenine elicited a peak plasma concentration (Cmax) of 28 µM, which was 4 and 18 times higher than with 4 (*p* = 0.0034) and 1 mg/kg (*p* = 0.0017), respectively. CN1 activity showed strong negative correlations with half-life (ρ = − 0.829; *p* = 0.0583), Cmax (r = − 0.938; *p* = 0.0372) and incremental area under the curve (r = − 0.825; *p* = 0.0433). Overall, balenine seems more resistant to CN1 hydrolysis resulting in better in vivo bioavailability, yet its degradation remains dependent on enzyme activity. Although a similar functionality as carnosine and anserine remains to be demonstrated, opportunities arise for balenine as nutraceutical or ergogenic aid.

## Introduction

Carnosine (β-alanyl-L-histidine), and its methylated analogues anserine (β-alanyl-N(π)-methyl-L-histidine) and balenine (β-alanyl-N(τ)-methyl-L-histidine) are histidine-containing dipeptides (HCD), abundantly present in excitable tissues of various species^1^. Both carnosine and anserine have been extensively explored as dietary ingredient and supplement regarding their potential health and performance enhancing properties^2–5^. Research on balenine’s bioactivity is less extensive, possibly because it is mainly found in species (marine mammals and reptiles) that do not contribute significantly to the human diet, in contrast to the high carnosine and anserine intake through pork/beef and poultry/fish, respectively^1,6^. Yet, some research on balenine has been emerging in the past years^7,8^, and we recently found balenine in human skeletal muscle^9^.

The chemical structure difference between the compounds is subtle, with no methylation (carnosine) or methylation of the imidazole group at the N(π) (anserine) or N(τ) (balenine) position (depicted in Fig. 2A). Still, cell culture and animal studies suggest the presence of similar but also some different properties for balenine as compared to carnosine and anserine. Balenine was found to have a higher antioxidant and iron-chelating capacity than carnosine and anserine^7,10–13^. In contrast, balenine lacks the ability to quench reactive aldehydes such as 4-hydroxy-2-nonenal (HNE) and acrolein^10^.

Translation of carnosine research from rodent disease models to humans is hampered by the highly active serum carnosinase (CN1) enzyme in human circulation, which is absent in rodents^14^. The CN1 enzyme is responsible for the rapid hydrolysis of HCDs in circulation immediately upon absorption. In several in vitro experiments, the hydrolysis rate of anserine is 30–50% of carnosine’s while balenine has an even lower rate, approximately 5–20%. This means that carnosine, anserine and balenine are respectively good, intermediate and poor substrates for the CN1 enzyme^15–17^. In vivo human experiments show a rapid degradation of carnosine upon absorption. Ingestion of carnosine elicited a small increase in plasma concentration, but only in people with low CN1 enzyme activity and with a very high dose of 60 mg/kg bodyweight^18,19^. Ingesting anserine, on the other hand, elicited dose-dependent peak concentrations of 0.5–3.1 µM. Increasing plasma anserine appeared to pose less of a challenge. Nevertheless, up to a dose of 10 mg/kg, measurable plasma anserine was mainly observed in people with low CN1 enzyme activity^18,20^. It was shown that carnosine and anserine are absorbed intact into the bloodstream, but their short elimination half-life and low to undetectable concentrations in the human systemic circulation may be a major limitation for translating their therapeutic potential. Therefore, as balenine was shown in in vitro studies to have a low hydrolysis rate and its functionality partly resembles carnosine’s and anserine’s, it might be an even better nutraceutical. Hence, it is necessary to gain a better insight into its oral bioavailability and pharmacokinetic (PK) properties.

The aim of this study was twofold: first, we tested balenine’s degradation rate in vitro and hypothesized that it would be slow as it was hypothesized not to be a suitable substrate for CN1. Secondly, an in vivo PK study was conducted in which absorption, distribution, metabolism and excretion of balenine was studied. We optimized and validated an ultra-high performance liquid chromatography tandem mass spectrometry (UHPLC-MS/MS) method to quantify HCDs at low concentrations with high accuracy and precision. Population pharmacokinetic analysis was performed to gain insight in the best possible dosing regimens for future studies.

## Results

### In vitro experiment

**Figure 1:**
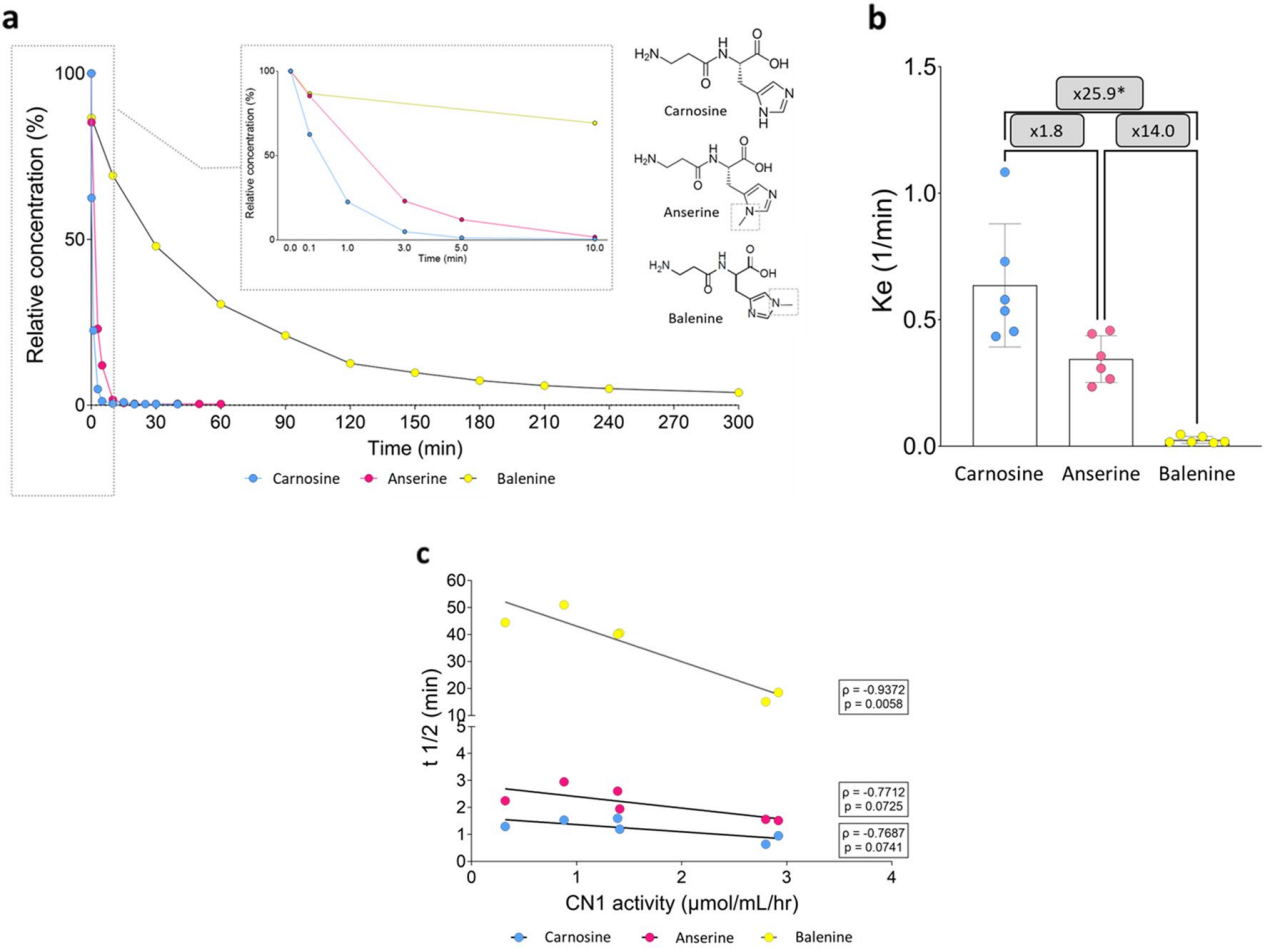
Mean breakdown profile of carnosine, anserine and balenine, relative to their starting concentration. The panel in the middle zooms in on the profile up to 10 min. Molecular structure of carnosine, anserine and balenine in which the methyl-group is indicated (**A**). The mean elimination rate constant (ke) of carnosine, anserine and balenine, and the factor difference between the dipeptides (**B**). Correlation between the half-life (t1/2) of the dipeptides in human plasma samples of different subjects and their respective CN1 enzyme activity (**C**). *p < 0.05.

Figure 1A shows the mean breakdown profile (relative to starting concentration) of the HCDs. Carnosine and anserine are rapidly hydrolysed upon addition to the heparin plasma and appear undetectable after 5 and 15 min, respectively. In contrast, balenine remains elevated above LOQ up to 5 h. The elimination constant, ke, presented a similar pattern (Fig. 1B). A Friedman test (*p* = 0.0001) showed a significantly lower ke for balenine (× 25.9; *p* = 0.0016) compared to carnosine. No significant differences were found between the ke of balenine and anserine (× 14.0; *p* = 0.250) or carnosine and anserine (× 1.8; *p* = 0.250). Half-life of carnosine (1.20 ± 0.36 min) was 29.1 times lower (*p* = 0.0044) than that of balenine (34.9 ± 14.6 min). Moreover, half-life of anserine (2.14 ± 0.58 min) was significantly different from carnosine (× 1.8; *p* = 0.0015) or balenine (× 16.3; *p* = 0.0044).

The correlation coefficient shows strong negative relations for the three HCDs between their half-life and CN1 activity, although only significant for balenine (carnosine: ρ = − 0.769; *p* = 0.0741; anserine: ρ = − 0.771; *p* = 0.0725; balenine: ρ = − 0.937; *p* = 0.0058) (Fig. 1C).

### In vivo pharmacokinetic experiment

**Figure 2:**
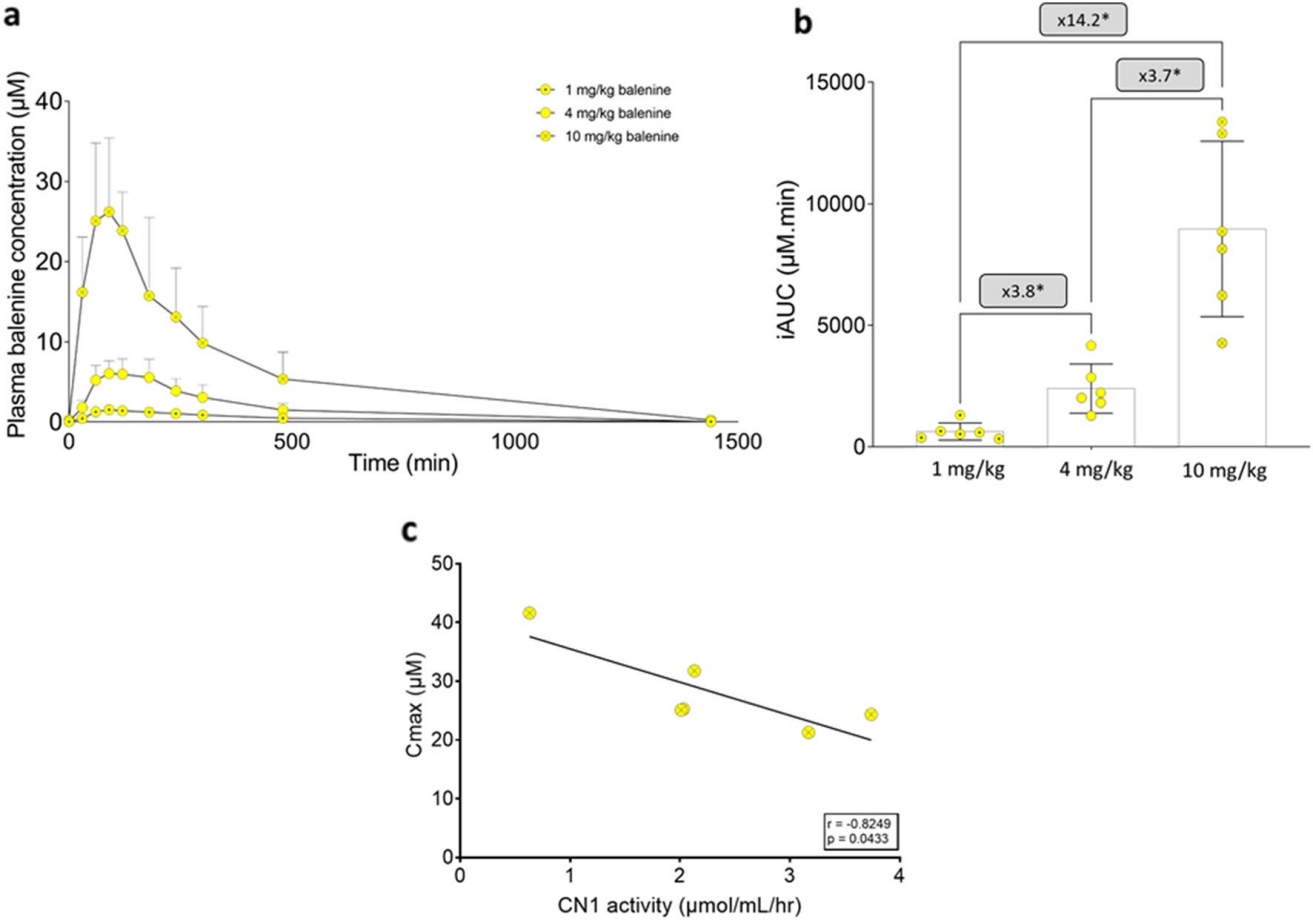
Mean + SD (n = 6) plasma balenine concentration time curve after a single ingestion of 1, 4 and 10 mg/kg of balenine (**A**). iAUC (n = 6) of balenine for the three different doses (**B**). Correlation between Cmax and CN1 activity in the 10 mg/kg dose (**C**). *p < 0.05.

Plasma balenine remained significantly increased up to 3 h after ingesting 4 mg/kg balenine (95% CI: 515–9860; *p* = 0.017) and up to 8 h following 10 mg/kg (95% CI = 444–9756; *p* = 0.02) (Fig. 2A). The incremental area under the plasma concentration time curve (iAUC) increased significantly (*p* = 0.001) with increasing doses. The iAUC was 3.8 times higher with 4 mg/kg compared to 1 mg/kg (95% CI = 856–2674; *p* = 0.003) and 3.7 times higher following 10 mg/kg compared to 4 mg/kg (95% CI = 2479–10,653; *p* = 0.010). A 14.2 fold increase was observed after ingestion of 10 mg/kg compared to 1 mg/kg (95% CI = 3913–12,749; *p* = 0.006) (Fig. 2B).

Similar to iAUC, Cmax significantly increased with increasing doses (*p* = 0.0005). The Cmax was 4.4 times higher with 4 mg/kg (7.1 ± 1.2 µM) compared to 1 mg/kg (1.6 ± 0.5 µM) (95% CI = 4.29–6.62; *p* < 0.0001) and 4 times higher following 10 mg/kg (28.2 ± 7.4 µM) compared to 4 mg/kg (95% CI = 11.4–31.0; *p* = 0.003). A 17.6 fold increase was observed after ingesting 10 mg/kg compared to 1 mg/kg (95% CI = 16.3–36.9; *p* = 0.002).

Since iAUC and Cmax in the 1 and 4 mg/kg dose correlated with body weight from which dose was determined, only the 10 mg/kg (fragmented dose) will be shown. For iAUC and Cmax, a significant negative correlation was found with CN1 enzyme activity (iAUC: r = − 0.938; *p* = 0.037, Cmax: r = − 0.825; *p* = 0.043; Fig. 2C) indicating that with higher activity, more balenine is hydrolysed. For time to peak concentration, no significant correlation was observed (r = − 0.559; *p* = 0.248).

Regarding urinary balenine excretion, after ingesting 1 mg/kg, 19.3 ± 13.1 mg is excreted, which was as expected 4 times lower than the 82.0 ± 40.5 mg after ingesting 4 mg/kg (95% CI: 23.3–102; *p* = 0.008) and the 176 ± 95.9 mg after ingesting 10 mg/kg (95% CI: 32.4–282; *p* = 0.024). These amounts correspond to 27.3%, 29.7% and 25.2% of the ingested dose with 1, 4 and 10 mg/kg, respectively. These percentages were negatively correlated with CN1 activity (1 mg/kg: r = − 0.941; *p* = 0.005; 4 mg/kg: r = − 0.888; *p* = 0.018; 10 mg/kg: r = − 0.896; *p* = 0.016).

### Population pharmacokinetic modelling of balenine

A 1-compartment model with sequential zero- and first-order absorption and linear elimination was identified as the best model to describe plasma concentration-time profiles of balenine. The zero-order input lasted 1.13 h for the 1 and 4 mg/kg doses and was shorter for the 10 mg/kg dose. It was followed by a fast first-order absorption (ka = 3.48/h, fixed to its last estimated value, or an absorption half-life of 0.2 h). The volume of distribution (V) was estimated at 126 L, and the total clearance (CL) at 19.6 L/h (1 and 4 mg/kg), which is low. The PK parameters of balenine were dose-proportionally between the two lowest doses, but increased more than dose-proportional beyond 4 mg/kg. This translated in a lower volume of distribution and CL for the 10 mg/kg dose level. More than half of the balenine dose was eliminated unchanged through the kidney (56.4%). Inter-occasion variability, such as random variability in subjects’ physiological parameters from one visit to the next or in the applied methods unrelated to the compound, was implemented on all parameters, and ranged from 0.11 to 0.38 (SD). Higher CN1 activity resulted in a significantly higher clearance, which is also reflected in the negative correlations with Cmax and iAUC, and a lower fraction excreted unchanged via the kidney. Pharmacokinetic parameters of the final model are summarized in Supplementary Table 1.

## Discussion

In the present study, in vitro stability of balenine in human plasma and its in vivo bioavailability was investigated. The experiments were conducted with a particular interest in the relationship with CN1 activity and in comparison to the parent molecule carnosine and the alternatively methylated derivative anserine.

To measure balenine with high accuracy and precision, we optimized and validated an UHPLC-MS/MS method. In short, no carry-over was observed LOQ was 0.05 µM and linearity was confirmed up to 10 µM. Precision and accuracy were assessed within and between runs and passed the FDA requirements for analytical method development^21^. So, this method is validated to measure balenine accurately.

Previous in vitro research has shown that balenine is a poorer substrate for CN1 than carnosine and anserine^15,16^. This is corroborated by our in vitro study. Herein, the half-life of balenine was around 30 min while that of carnosine and anserine was only 1 and 2 min, respectively. Additionally, the reported rate of hydrolysis of balenine (relative to carnosine) was 3.81%^16^ and 8%^15^. Our study confirms these results as the elimination rate constant of balenine was 3.86%. In addition to this confirmation of earlier findings, we hypothesized balenine to disappear more slowly from the systemic circulation with no relationship to CN1 activity, as compared to carnosine and anserine. However, elimination half-life and constant of balenine in vitro were negatively correlated to CN1 enzyme activity. In plasma with the highest CN1 activity, balenine had a half-life of 15 min for balenine while balenine in the plasma with the lowest enzyme activity had a threefold higher half-life. This indicates a strong effect of CN1 activity on elimination rate, which may explain high inter-individual differences in PK profile when balenine is acutely ingested. In addition to the in vitro findings, peak plasma concentration and iAUC following 10 mg/kg of balenine were also correlated with CN1 activity. This indicates that, although the CN1 enzyme has very low affinity towards balenine, its stability remains dependent on CN1 activity. These results counter our initial hypothesis.

In order to implement acute balenine ingestion as possible nutraceutical or performance enhancing supplement, its bioavailability after ingestion needs to be established. The main findings of the PK study are the high stability of balenine, as opposed to carnosine and anserine. When looking more closely to iAUC and peak plasma concentration of balenine, a non-linear higher increase in plasma balenine concentration is observed with higher doses. It is speculated that this results from a saturation of CN1, meaning that all binding sites for hydrolysis are occupied at a certain concentration. From then on, plasma balenine concentration increases exponentially^20^.

**Figure 3:**
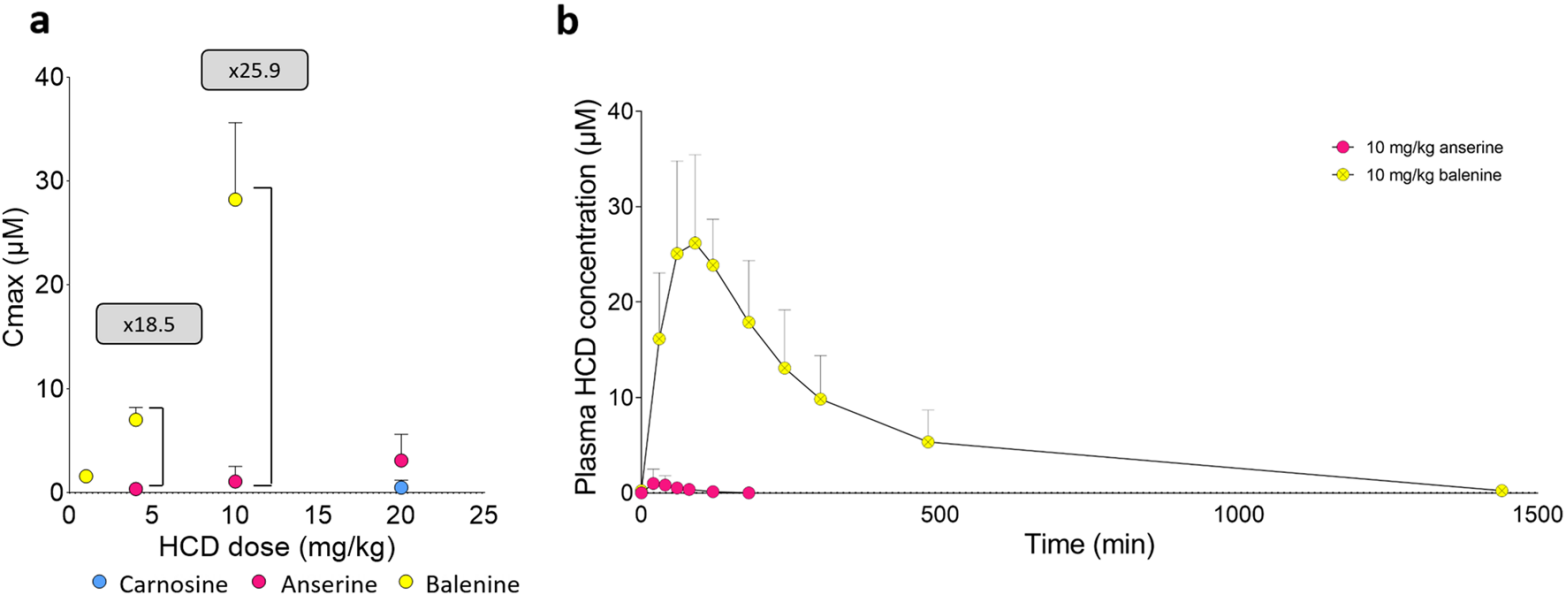
Comparison of Cmax values of carnosine (n = 12)^22^, anserine (n = 7)^20^ and balenine (n = 6) according to the doses ingested (**A**). Plasma concentration time profile following ingestion of 10 mg/kg anserine^20^ or balenine (**B**). Mean ± SD are shown.

**Figure 4:**
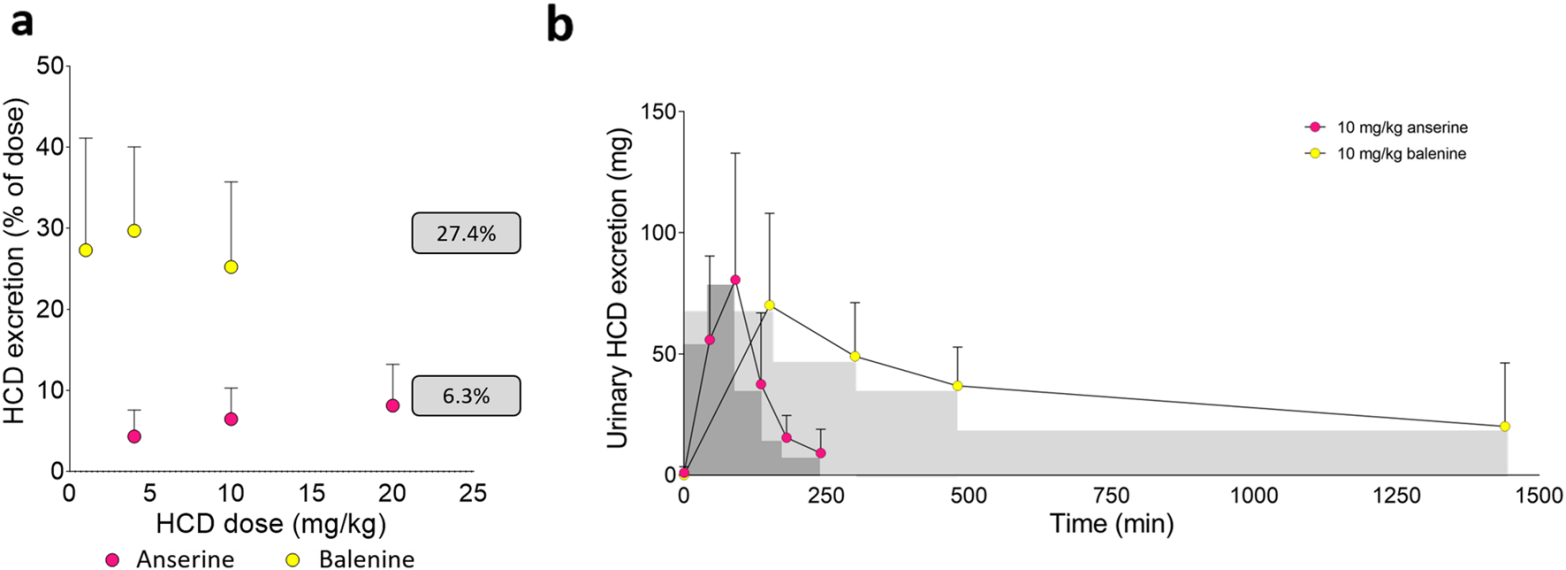
Comparison of the percentage of the dose anserine (n = 7)^20^ and balenine (n = 6) that is excreted intact in the urine (**A**). Urine concentration time profile following ingestion of 10 mg/kg anserine^20^ or balenine (**B**). Mean ± SD are shown.

Since carnosine and anserine PK data is also available from previously published studies from our lab^20,22^, all data for oral ingestion of 1, 4, 10 or 20 mg/kg of carnosine, anserine or balenine in isolation, were integrated in Fig. 4. Carnosine was barely measurable, even after ingesting the highest dose of 20 mg/kg^22^. Cmax for anserine was 0.54 µM and 1.10 µM for the 4 and 10 mg/kg dose^20^ but these concentrations were 18.5 and 25.9-fold higher when a same amount of balenine was ingested (Fig. 3A). Additionally, when comparing the plasma time-course following ingestion of 10 mg/kg of either anserine or balenine, a very large difference in plasma iAUC is observed (Fig. 3B). For anserine, the iAUC was 66.7 µM.min while for balenine 8960 µM.min, which is a 136-fold difference. Overall, this shows that pure balenine is absorbed intactly with a much higher bioavailability than both carnosine and anserine, again indicating a much lower susceptibility to CN1 hydrolysis.

When urinary dipeptide excretion is compared, the average excreted percentage of the dose is 6.3% for anserine but 27.4% for balenine (Fig. 4A). Interestingly, when comparing ingestion of 10 mg/kg of anserine and balenine, almost equal amounts of balenine are excreted in urine in the first hours following ingestion, while the plasma concentration of balenine, and thus the amount filtered, is a lot higher (Fig. 4B). A possible explanation relates to the affinity of HCDs to the human peptide transporter 2 (PEPT2) in the proximal kidney tubule. PEPT2 contributes to the reabsorption of filtered peptides^1^. Anserine is a high-affinity ligand for PEPT2^23^, which could result in high reabsorption from the filtrate in the kidney. The higher reabsorption in our study might indicate that balenine is an even better substrate for PEPT2. Indeed, some preliminary molecular dynamics experiments showed a possibility for improved tubular balenine reabsorption compared to anserine (Prof. Giulio Vistoli; personal communication). This higher reabsorption of balenine may also contribute to its increased bioavailability.

Carnosine was shown, mostly in rodents, to affect various pathologies such as multiple sclerosis^24^, Alzheimer’s^25,26^, type II diabetes^27,28^, metabolic syndrome^29^ and many other (reviewed by Artioli et al.^2^). Although carnosine supplementation in humans occasionally appears beneficial^30–32^, translating its full potential remains a challenge, mainly because of the highly active CN1 enzyme in human circulation which is absent in rodents^14^. Balenine’s high stability and bioavailability might be a way to overcome this translation difficulty. One prerequisite is that balenine’s functionality should resemble that of carnosine. Although research into this topic is limited, it appears promising with evidence of an antioxidant capacity^7,11–13^, reaction with cytotoxic carbohydrate-derived aldehydes^33^ and an effect on learning and memory in an Alzheimer’s disease mouse model^34^. Future human studies should investigate whether balenine is indeed a better nutraceutical than carnosine and anserine, because of its superior stability and bioavailability. In addition to these possible health effects, balenine supplementation may also hold promise for ergogenic (sport performance) purposes. A negative correlation between CN1 activity and performance improvement indicates that bioavailability of the dipeptide is a prerequisite for improving performance^35^. Balenine could be a better alternative to carnosine and anserine supplementation in a sports setting as well.

Balenine is the first endogenous HCD that shows such good stability and bioavailability in humans. The fact that it is a naturally occurring compound in our diet makes the toxicology profile and therapeutic potential highly feasible. In addition, pharmacological variants of carnosine have been developed, such as Trolox-carnosine^36^, D-carnosine octylester^37^ and carnosinol^29^. The latter is a reduced carnosine derivative that is resistant to CN1 and has shown promise to be used in the treatment of metabolic diseases of obesity and diabetes.

To conclude, this research corroborated previous findings stating that balenine is significantly more stable in vivo than anserine and carnosine. This was reflected in vivo in a higher peak plasma concentration and iAUC after supplementing with lower doses of balenine, compared to carnosine and anserine. Moreover, balenine stability appears to be dependent on serum CN1 enzyme activity. Since balenine has a somewhat similar functionality as carnosine and anserine, this might generate much better opportunities for developing balenine as nutraceutical or ergogenic aid, when translating animal research to humans.

## Methods

This paper includes an in vitro and in vivo pharmacokinetics (PK) experiment to determine absorption, degradation and elimination of balenine, carnosine and anserine in plasma with varying CN1 enzyme activity. All subjects gave their written informed consent and the study was approved by the Local Ethics Committee of the Ghent University Hospital (BC07532). It was performed in accordance with the standards of ethics outlined in the Declaration of Helsinki.

Pure, chemically synthesized carnosine and anserine were kindly provided by Flamma S.p.a. (Chignolo d’Isola, Bergamo, Italy) and the balenine powder by NNB Nutrition (Frisco, Texas, USA).

### In vitro experiment

Fasted venous heparin plasma was collected from 6 subjects (5 males and 1 female) with a wide range in CN1 enzyme activity (0.32–2.92 µmol/mL/h with a mean value of 1.62 µmol/mL/h). Subjects were asked to refrain from alcohol, meat and fish the day before the sampling. In separate tubes, a final concentration of 20 µM of carnosine, anserine and balenine were added to the plasma, which was kept at 37 °C. On different time points (Table 1), plasma was deproteinized with 1:11 sulfosalicylic acid (SSA, 35% solution) to inhibit further hydrolysis. Samples were centrifuged for 5 min at 16,000 g and supernatant was stored at − 20 °C until further UHPLC-MS/MS analysis.

**Table 1:** Overview of the time points on which plasma was deproteinized (time in minutes after adding carnosine, anserine and balenine).

Time points (min)	t1	t2	t3	t4	t5	t6	t7	t8	t9	t10	t11
Carnosine	0	1	3	5	10	15	20	25	30	40	/
Anserine	0	3	5	10	15	20	30	40	50	60	/
Balenine	0	10	30	60	90	120	150	180	210	240	300

### In vivo PK experiment

Since this study is the first to supplement pure, chemically synthesized balenine to humans, a thorough safety evaluation was conducted by Vedic Lifesciences (Mumbai, India) (190,911/WGI/PC). Seven female Sprague Dawley rats (8–12 weeks old) were acutely supplemented with increasing doses of balenine up to 2000 mg/kg. Following treatment, a 2 week observation period with twice daily examination for morbidity and mortality was performed. No pathological changes were observed in any animal. It was concluded that a dose of 2000 mg/kg balenine was safe to use in the following experiments.

Six subjects (3 males and 3 females) participated in this PK study in which three increasing doses were tested. For the third dose, one female was replaced, due to pregnancy, by another woman with similar CN1 activity. Their mean (n = 7) weight, height and age were 66.4 ± 10.1 kg, 171 ± 12 cm and 25.4 ± 1.7 years. CN1 activity ranged between 0.63 and 3.74 µmol/mL/h with a mean of 2.43 µmol/mL/h. Since this was the first time pure balenine was ingested by human volunteers, no dose randomization, but a gradual increase in dose was opted for. On the first test day, subjects received 1 mg/kg bodyweight of balenine and 4 mg/kg the following test day. Test days were separated by at least two weeks to ensure complete return to baseline balenine concentration. After analysing these samples, a third test day was added in which 10 mg/kg in a fragmented dose was provided to exclude the effect of bodyweight on balenine concentration in plasma (Stautemas et al. 2019). This means that half of the dose was administered as 5 mg/kg (weight-relative part) and the other half was an absolute dose based on the mean weight of the subjects of the third test day (341 mg; fixed part).

**Figure 5:**
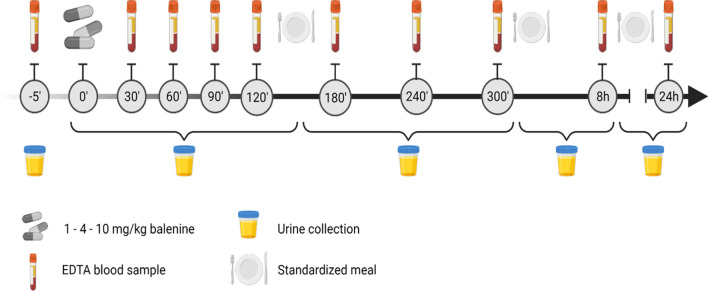
Timeline of a test day (Figure created with BioRender.com).

The day before a test day, subjects were asked to refrain from meat and fish. Upon arrival, a catheter was inserted in an antecubital vein and a fasted ethylenediaminetetraacetic acid (EDTA, 4 mL) and serum sample (3.5 mL, to determine CN1 activity) were collected. Then they were asked to empty their bladder, after which the supplement, being 1, 4 or 10 mg/kg balenine in gelatine capsules was ingested. The following 2 h, every 30 min an EDTA blood sample was taken. Then after 3, 4, 5, 8 and 24 h, another sample was withdrawn. Urine was collected after 150 min, 5, 8 and 24 h. The subjects arrived in the lab and remained fasted until 2 h after ingesting the supplement. Subjects remained in the lab until the post 8 h blood sample, and returned the following morning for the post 24 h sample. Throughout the test day, food intake was standardized (lacto-ovo-vegetarian diet) ensuring minimal HCD intake for 24 h following supplement. Figure 5 gives a schematic overview of a test day.

Plasma samples were collected in pre-cooled (4 °C) EDTA tubes. Upon withdrawal, plasma was immediately deproteinized with 1:11 SSA, centrifuged (5 min, 16 000 g) and stored at − 20 °C.

### Sample preparation

Extraction of plasma and urine for UHPLC-MS/MS analysis went as follows: 150 µL of deproteinized plasma/urine (not deproteinized) was added to 215 µL of acetonitrile containing 1% formic acid, 10 µL of internal standard (IS) (carnosine-D4) and 25 µL ultrapure water or standard (calibration curve and quality control samples (QC)). Then samples were vortexed and centrifuged (15 min, 4 °C, 15,000 g). Supernatant was used for further analyses.

In the in vitro study, supernatant was diluted 40 times before injection. In the PK study, plasma samples from the 10 mg/kg condition and baseline urine samples were diluted 100 times. All other urine samples were diluted 1000 times. Dilutions were conducted with acetonitrile containing 1% formic acid.

Pooled heparin (in vitro study) and EDTA (in vivo study) plasma, and urine were used to prepare calibration curve and QC samples (identical extraction protocol as samples). For quantification, the following concentration ranges were used: in vitro study and 1 and 4 mg/kg condition: 0–25 µM with QC at 0.05, 0.5 and 5 µM; 10 mg/kg condition: 0–100 µM with a fourth QC of 50 µM; urine: 0–60 µM with QC at 0.05, 0.5, 5 and 50 µM.

### UHPLC-MS/MS conditions

Samples were analysed by an in-house developed and validated UHPLC-MS/MS method. Carnosine-D4, was purchased from Sanbio B.V. (Uden, The Netherlands) and acetonitrile, formic acid, ammonium formate and 25% ammonia solution from Merck (VWR International, B.V., Amsterdam, The Netherlands).

Analyses were performed on a UHPLC-MS/MS platform consisting of an Acquity H-Class Quaternary Solvent Manager and Flow-Through-Needle Sample Manager with temperature controlled tray (8 °C) and column oven (60 °C). Chromatographic separation was achieved on an Acquity UPLC BEH amide column (2.1 mm × 100 mm, 1.7 µm) with an Acquity amide Vanguard pre-column. The system operated at a flow rate of 0.3 mL/min with solvent A (ultrapure water), solvent B (acetonitrile) and solvent C (200 mM ammonium formate with 0.04% ammonia) with the gradient shown in Table 2. The UPLC column effluent was interfaced to a Xevo TQ-XS® MS/MS system (plasma samples) and Xevo TQ-S® (urine samples), equipped with an electrospray ionization (ESI) probe operating in positive ion mode. Further MS settings included desolvation gas flow rate (15 L/h), desolvation temperature (600 °C), cone gas flow rate (800 L/h), source temperature (150 °C) and capillary voltage (0.75 kV). Transition details are depicted in Table 3. Instrument parameters were optimised by direct infusion of working solution of 100 ng/mL of carnosine, anserine, balenine and IS at a flow rate of 10 µL/min and in combination with the mobile phase (15% A, 80% B, 5% C) at a flow rate of 300 µL/min. The detector operated in multiple reacting monitoring mode, scanning the two most intense transitions of the HCDs. Masslynx software 4.2 was used for instrument control and data extraction. Hardware and software were purchased from Waters (Milford, MA, USA).

**Table 2:** Chromatographic gradient for HCD quantification.

Time (min)	%A	%B	%C
0	15	80	5
1	15	80	5
7	45	50	5
8	45	50	5
8.01	15	80	5
12	15	80	5

**Table 3:** Mass spectrometry transitions for HCD quantification.

Transitions	Ion	m/z precursor ion	m/z product ion	Cone voltage (V)	Collision energy (eV)
CARNOSINE	Quantification	227.2	110.1	35	20
	Confirmation	227.2	156.1	35	13
ANSERINE	Quantification	241.2	109.1	30	23
	Confirmation	241.2	170.1	30	15
BALENINE	Quantification	241.2	124.1	30	24
	Confirmation	241.2	170.1	30	15
CARNOSINE-D4	Quantification	231	110.1	30	22
	Confirmation	231	156	30	15

*m/z* Mass to charge ratio,.

### UHPLC-MS/MS method validation for the quantification of balenine

This method was validated on three separate days according to the US FDA guidelines for bio-analytical method validation^21^. The following parameters were considered: selectivity, linearity, within- and between- run precision and accuracy, and carry-over effect.

Plasma samples, withdrawn after two days of lacto-ovo-vegetarian diet, showed a small endogenous peak at the retention time of balenine. Nevertheless, the method is considered selective since the chromatographic peak area of this sample is on average 5.79% of the area of balenine at 0.05 µM (limit of quantification; LOQ). This is lower than 20% at LOQ level, as indicated by the FDA guidelines. Linearity of the calibration curve was good from 0.05 µM to 10 µM. The equation y = ax + b, with a the slope and b the intercept (mean ± SD), was y = 0.00358 (± 0.00038)x + 0.00577 (± 0.00818) with r = 0.9974 (± 0.0011) and a goodness of fit of 6.49 (± 0.98)%. No carry-over was observed following the highest QC and calibration samples. Within and between-run precision and accuracy were evaluated by analysing three concentrations (0.05–0.5–5 µM) six and two times, respectively, on three separate occasions. Results are summarized in Table 4.

**Table 4:** Results of the within and between-run precision and accuracy evaluation for the analysis of balenine in plasma.

Theoretical concentration of BAL (µM)	Mean concentration ± SD (µM)	Precision, RSD (%)	Accuracy (%)
0.05^a^	0.051 ± 0.001	2.54	2.97
0.05^b^	0.048 ± 0.007	13.72	− 3.94
0.5^a^	0.45 ± 0.02	5.34	− 9.69
0.5^b^	0.46 ± 0.08	17.67	− 8.65
5^a^	4.46 ± 0.25	5.72	− 10.85
5^b^	4.49 ± 0.36	8.06	− 10.26

^a^within-run analysis (n = 6; on 3 separate days); ^b^between-run analysis (n = 2; on 3 separate days); *SD* Standard deviation, *RSD* Relative standard deviation.

### Serum carnosinase (CN1) activity

CN1 activity was quantified by fluorometric determination of histidine following carnosine addition (based on Teufel et al., 2003). The reaction was initiated by adding carnosine to the serum (final concentration of 3.33 mM carnosine) and stopped after 10 min incubation at 37 °C by adding trichloroacetic acid (final concentration of 150 mM TCA). For controls, trichloroacetic acid was added before carnosine. After centrifugation (15 min, 16000 g), 10 µl supernatant was added to 190 µl of an OPA-NaOH mixture (4000 µl incomplete ophtaldehyde, with 0.2% 2-mercaptoethanol, and 15 ml of 4 M sodium hydroxide solution) and fluorescence was determined after 40 min (excitation: 360 nm and emission: 465 nm). All chemicals were purchased from Sigma-Aldrich (Milan, Italy).

### Calculations and statistical analysis

HCD concentration in the in vitro experiment was calculated relative to the starting concentration. Elimination constant (ke) and elimination half-life were calculated based on a monophasic decay profile. Incremental area under the plasma concentration time curve (iAUC) was calculated using the trapezoidal method in the PK study. A normality check was performed using Shapiro-Wilk’s test. In case of normally distributed data, a mixed-effects model and Pearson correlation was used. Skewed data were analysed with Friedman tests and Spearman rank correlations. Values are presented as mean/median ± standard deviation/inter quartile range and 95% confidence intervals (95% CI). Significance is assumed at *p* ≤ 0.05. Graphpad Prism (version 9; GraphPad Software, San Diego, California) was used for statistical analyses.

Plasma and urine balenine concentrations (in vivo) were analysed using a nonlinear mixed-effects modelling approach as implemented in the Monolix software (Version 2021R1; Lixoft, Antony, France). Plasma concentrations were described using a 1-compartmental model, with sequential zero- and first-order absorption and linear elimination. Cumulative urine concentrations were fitted simultaneously with plasma concentrations. Inter-occasion variability was also implemented and assumed to follow a lognormal distribution. Residual variability was described using a combined error model, consisting of an additive and a proportional component. The following covariates were tested: sex, age, weight, CN1 activity and dose. Model evaluation was done based on standard goodness-of-fit metrics (objective function value, standard errors) and plots.

## Supplementary Information

Supplementary Information.

## Data Availability

Raw data were generated at the Sports Science Laboratory, Jacques Rogge, Watersportlaan 2, 9000 Ghent, Belgium. Derived data supporting the findings of this study are available from the corresponding author WD on request.
