# Supplementary material for: Acute balenine supplementation in humans as a natural carnosinase-resistant alternative to carnosine
Source: Sci Rep. 2023 Apr 20;13:6484. doi: 10.1038/s41598-023-33300-1 (PMC10119279; doi:10.1038/s41598-023-33300-1)
Supplement: Supplementary file 1 — Supplementary Information. [file 41598_2023_33300_MOESM1_ESM.docx]

Supplementary material

Table 1 – Estimates of the population pharmacokinetic parameters

| **FIXED EFFECTS** | | | |
| --- | --- | --- | --- |
|  | **Value** | **Standard error (SE)** | **Relative SE (%)** |
| **Absorption constant (ka) (1/h)** | 3.48 |  |  |
| **Volume (V) (L)** | 126 | 6.04 | 4.79 |
| **Clearance (CL) (L/h)** | 19.6 | 2.12 | 10.8 |
| **Fraction excreted in urine (pu)** | 0.56 | 0.036 | 6.27 |
| **dT1** | 1.13 | 0.151 | 13.3 |
| **β V - dose** | -0.38 | 0.072 | 19.3 |
| **β Cl - dose** | -0.42 | 0.086 | 20.3 |
| **β Cl - CN1** | 0.25 | 0.042 | 16.7 |
| **β pu - CN1** | -0.54 | 0.056 | 10.3 |
| **β dT1 - dose** | -0.74 | 0.23 | 31.1 |
| **SD of the RANDOM EFFECTS** | | | |
| **γ ka** | 0.38 |  |  |
| **γ V** | 0.11 | 0.032 | 28.4 |
| **γ Cl** | 0.15 | 0.031 | 20.2 |
| **γ pu** | 0.22 | 0.042 | 19.4 |
| **γ dose** | 0.36 | 0.083 | 22.7 |
| **ERROR MODEL PARAMETERS** | | | |
| **a (plasma)** | 0.038 | 0.0037 | 9.92 |
| **b (plasma)** | 0.12 | 0.013 | 10.8 |
| **a (urine)** | 0.075 | 0.012 | 16.3 |
| **b (urine)** | 0.088 | 0.0099 | 11.2 |

Β values indicate covariate effects
